# Analysis of prognostic factors and construction of prognostic models for invasive lobular carcinoma of the breast

**DOI:** 10.17305/bb.2024.10578

**Published:** 2024-12-01

**Authors:** Lin Cheng, Jianlin Wang, Liming Tang

**Affiliations:** 1 Department of Breast Surgery, The Affiliated Changzhou Second People’s Hospital of Nanjing Medical University; Changzhou Medical Center, Nanjing Medical University; Postdoctoral Station of Changzhou Second People’s Hospital, Changzhou, China; 2 Department of Radiotherapy, The Affiliated Changzhou Second People’s Hospital of Nanjing Medical University, Changzhou Medical Center, Nanjing Medical University, Changzhou, China; 3 Department of Gastrointestinal Surgery, The Affiliated Changzhou Second People’s Hospital of Nanjing Medical University; Changzhou Medical Center, Nanjing Medical University; Postdoctoral Station of Changzhou Second People’s Hospital, Changzhou, China

**Keywords:** Invasive lobular carcinoma (ILC), Surveillance, Epidemiology, and End Results (SEER), nomogram, prognosis, independent risk factors

## Abstract

Invasive lobular carcinoma (ILC) and invasive ductal carcinoma (IDC) account for most cases of breast cancer. However, there is ongoing debate about any potential variations in overall survival (OS) between ILC and IDC. This study aimed to compare survival between IDC and ILC, identify prognostic factors for ILC patients, and construct a nomogram for predicting OS rates. This retrospective cohort analysis utilized data from the Surveillance, Epidemiology, and End Results (SEER) Cancer Database. Patients diagnosed with ILC and IDC between 2000 and 2019 were enrolled. To minimize baseline differences in clinicopathological characteristics and survival outcomes, a propensity score matching (PSM) method was used. Data from the multivariate Cox regression analyses were used to construct a predictive nomogram for OS at one, three, and five years, incorporating all independent prognostic factors. Following the PSM procedure, patients with ILC exhibited a better prognosis compared to those with IDC. TNM stage, age >70, radiotherapy, surgery, estrogen receptor (ER), progesterone receptor (PR), and human epidermal growth factor receptor 2 (HR−/HER2+) subtype were identified as independent factors for OS in ILC patients. Surgery and radiotherapy effectively reduced the risk of death, while chemotherapy did not demonstrate the same benefit. This model could support clinicians in evaluating the prognosis of ILC for decision making and patient counseling.

## Introduction

Invasive lobular carcinoma (ILC) is the second most commonly observed histological subclass of invasive breast carcinoma, accounting for approximately 5%–15% of cases [1–4]. In contrast to patients with the foremost subclass, invasive ductal carcinoma (IDC), women with ILC have a higher likelihood of positive lymph nodes, advanced histologic stage, and larger tumor sizes. They are also more likely to test positive for hormone receptors [5–7]. Accurate prognosis evaluations are crucial in making therapy decisions for breast cancer. Incorrect predictions can result in unwanted management for patients with a better prognosis and inadequate management for high-risk ones. Currently, most decisions on ILC treatment are based on clinical trials emphasizing IDC. This probably explains why guidelines from the National Comprehensive Cancer Network (NCCN) and the St Gallen International Expert Consensus remain advocating the ILC management with identical paradigms to IDC. Nonetheless, ILC has distinct characteristics, which is now widely recognized as a unique disease event. As suggested by growing clinical evidence, a “one-size-fits-all” approach to the entire invasive breast carcinomas is undesirable for particular subclasses like ILC. There are controversial results concerning the prognosis of ILC compared to IDC, with the prognosis of ILC reported as worse [8], no different [9, 10], and even better [11] than for IDC. The question of whether there are differences in overall survival (OS) and disease-free survival (DFS) between these two carcinoma subtypes remains disputable. The difference might be related to the number of patients, clinicopathological characteristics, and different databases.

Therefore, it is necessary to conduct further comparisons of survival between IDC and ILC using large databases and identify prognostic indicators specifically for patients suffering from ILC. The American Joint Committee on Cancer (AJCC) staging paradigm is traditionally adopted for evaluating the cancer patient prognosis, where the local (T), regional (N), and distant (M) extents of cancer are considered [12]. Nevertheless, patients with identical AJCC stages have still been observed to have greatly varying prognoses. That is because apart from the T, N, and M stages, a few clinicopathological characteristics can also impact the carcinoma patients’ prognosis [13]. Breast cancer prognosis may be influenced by factors, such as age, race, size of tumor, as well as statuses of human epidermal growth factor receptor-2 (HER2), estrogen receptor (ER), and progesterone receptor (PR). 

There has been extensive application of reliable tools called nomograms in oncological practice, which help quantitatively predict outcome probabilities in individual patients. Numerous studies have found that nomograms offer higher predictive accuracy than the AJCC staging system [14]. However, no nomogram has been published for the OS estimation in patients with ILC. One of the difficulties in validating prognostic and forecast diagnostics of ILC lies in an extended period of time from diagnosis to recurrence/recrudesce, making it challenging to obtain funding for and track prospective studies. This is further supported by occasionally conflicting data concerning if ILC or IDC leads to a worse prognosis with the progression of time. We eagerly anticipate progress in the field, as it is expected to benefit patients greatly. Our current work attempted to make a survival comparison of ILC against IDC, identify prognostic factors for ILC patients, and formulate a nomogram for OS rate forecasting.

## Materials and methods

### National Cancer Institute’s Surveillance, Epidemiology, and End Results (SEER) database

Our study utilized information from the updated May 2022 version of SEER, which includes demographic statistics, tumor characteristics, nodal stage, surgical details, vital status, and follow-up records from 18 different geographic regions. This database contains data from over three million patients, representing approximately 28% of the US population. Quality control measures are strictly enforced, with an error rate of less than 5% [15].

In this study, we diagnosed patients with IDC based on the International Classification of Diseases (ICD) 8500/3 histological code, and patients with ILC based on the 8520/3 code. We obtained SEER cancer statistics and treatment details, including surgery, chemotherapy, and radiation therapy, with authorization. As no personal patient information was involved, explicit consent was not necessary.

### Case selection

Between 2000 and 2019, we initially identified 200,192 women with IDC and 23,862 women with ILC who met the following criteria: women aged 18–90 with primary, unilateral breast cancer and confirmed laterality, and diagnosed with either ductal or lobular carcinoma. Detailed information on tumor grade, TNM stages, hormone receptor (ER and PR) status, treatment, and survival data were collected, as these are recognized indicators that may impact breast cancer prognosis [16]. In older patients (≥70 years), breast cancer may have particular significance [7, 8]. To ensure comparability between the two groups, propensity score matching (PSM) was employed, resulting in a final cohort of 47,724 patients, with 23,862 IDC patients and 23,862 ILC patients. To account for potential treatment differences and ensure consistent follow-up, we focused on the research period from 2000 to 2019, with the cutoff date being December 31, 2019. Tumor and nodal staging classification followed the AJCC staging paradigm for breast cancer, where the 6th edition guidelines were adopted for women diagnosed before 2009, and the 7th edition guidelines were adopted for women diagnosed between 2010 and 2019. Additionally, cases with poorly differentiated, undifferentiated, and anaplastic grades were classified as grade III. The focus of our analysis was on tumors with either pure lobular or pure ductal histology, excluding those with mixed ductal and lobular histology to maintain homogeneity within the groups.

### Statistical analysis

Disparities in characteristic variables between ILC and IDC were compared by the X^2^ test. The Cox regression model was utilized to examine the multivariate correlation of tumor characteristic variables with survival outcomes. A significance level of 0.05 was set to determine statistical significance. The survival endpoint in this study was OS, which was assessed by the Kaplan–Meier approach. OS referred to the period from the breast carcinoma confirmation to the mortality due to any cause. The log-rank test was used to determine the hazard ratio (HR) and corresponding 95% confidence interval (CI) for OS. The PSM technique was employed for lowering the baseline disparities in clinicopathological traits and survival prognoses. ILC and IDC patients were matched 1:1 based on age, race, laterality, primary site, surgery, TNM phase, subtype, radiation, chemotherapy, as well as statuses of HER2, ER, and PR. The PSM method was calculated through the “MatchIt” package in R software (version 3.6.2, Synergy Software, Inc., Essex Junction, VT, USA).

We randomly divided our eligible patients into training and validation sets in a 7:3 ratio. The training set was used to create a nomogram, while the validation set served for internal validation. Based on the results of the multivariate analysis, we established a nomogram that can predict the OS rates for one, three, and five years. To evaluate the efficacy of the nomogram, we used the C-index and a receiver operating characteristic (ROC) graph to assess its ability to distinguish between outcomes. The consistency between predicted probabilities and actual outcomes was determined through calibration graphs. We used bootstrapping with 1000 resamples to evaluate both calibration and discrimination. Decision curve analysis (DCA) plots were utilized to evaluate the practicality and benefits of the nomogram. Statistical analysis was carried out using SPSS software (version 22, SPSS Inc., Chicago, USA). A *P* value of less than 0.05 was deemed as statistically significant [17].

**Table 1:** Comparison of clinical characteristics between ILC and IDC groups in unmatched population

	**IDC, *n* (%) *N* ═ 200,192**	**ILC, *n* (%) *N* ═ 23,862**	***P***
*Age*			
≤ 35	4135 (2.1)	62 (0.3)	<0.001
> 35, ≤ 70	142470 (71.2)	15466 (64.8)	
> 70	53587 (26.8)	8334 (34.9)	
*Race*			
White	41206 (20.6)	3388 (14.2)	<0.001
Non-white	158986 (79.4)	20474 (85.8)	
*Laterality*			
Bilateral	4 (0.0)	1 (0.0)	1
Left/right	200188 (100.0)	23861 (100.0)	
*Primary Site*			
Center	62513 (31.2)	8032 (33.7)	<0.001
Upper	107384 (53.6)	12771 (53.5)	
Lower	30295 (15.1)	3059 (12.8)	
*Surgery*			
No	11376 (5.7)	1102 (4.6)	<0.001
Breast conserving	115955 (57.9)	11618 (48.7)	
Mastectomy	72861 (36.4)	11142 (46.7)	
*AJCC_Stage*			
I	109090 (54.5)	10698 (44.8)	<0.001
II	65855 (32.9)	8817 (36.9)	
III	18619 (9.3)	3503 (14.7)	
IV	6628 (3.3)	844 (3.5)	
*AJCC_T*			
1	126092 (63.0)	12249 (51.3)	<0.001
2	59085 (29.5)	8066 (33.8)	
3	9258 (4.6)	3048 (12.8)	
4	5757 (2.9)	499 (2.1)	
*AJCC_N*			
0	136482 (68.2)	15377 (64.4)	<0.001
1	49319 (24.6)	5946 (24.9)	
2	9286 (4.6)	1360 (5.7)	
3	5105 (2.6)	1179 (4.9)	
*AJCC_M*			
0	193539 (96.7)	23015 (96.5)	0.069
1	6653 (3.3)	847 (3.5)	
*Subtype*			
HR−/HER2−	25875 (12.9)	343 (1.4)	<0.001
HR−/HER2+	10098 (5.0)	109 (0.5)	
HR+/HER2−	141151 (70.5)	22361 (93.7)	
HR+/HER2+	23068 (11.5)	1049 (4.4)	
*ER_Status*			
Negative	38352 (19.2)	488 (2.0)	<0.001
Positive	161840 (80.8)	23374 (98.0)	
*PR_Status*			
Negative	58891 (29.4)	3972 (16.6)	<0.001
Positive	141301 (70.6)	19890 (83.4)	
*HER2_Status*			
Negative	167026 (83.4)	22704 (95.1)	<0.001
Positive	33166 (16.6)	1158 (4.9)	
*Radiation*			
No	95117 (47.5)	11734 (49.2)	<0.001
Yes	105075 (52.5)	12128 (50.8)	
*Chemotherapy*			
No	115238 (57.6)	16558 (69.4)	<0.001
Yes	84954 (42.4)	7304 (30.6)	

ILC: Invasive lobular carcinoma; IDC: Invasive ductal carcinoma.

**Table 2:** Comparison of clinical characteristics between ILC and IDC groups in matched population

	**IDC, *n* (%) *N* ═ 200,192**	**ILC, *n* (%) *N* ═ 23,862**	***P***
*Age*			
≤ 35	96 (0.4)	62 (0.3)	0.024
> 35, ≤ 70	15480 (64.9)	15466 (64.8)	
>70	8286 (34.7)	8334 (34.9)	
*Race*			
White	3415 (14.3)	3388 (14.2)	0.734
Non-white	20447 (85.7)	20474 (85.8)	
*Laterality*			
Bilateral	1 (0.0)	1 (0.0)	1
Left/right	23861 (100.0)	23861 (100.0)	
*Primary Site*			
Center	8011 (33.6)	8032 (33.7)	0.828
Upper	12747 (53.4)	12771 (53.5)	
Lower	3104 (13.0)	3059 (12.8)	
Surgery			
No	1126 (4.7)	1102 (4.6)	0.871
Breast conserving	11614 (48.7)	11618 (48.7)	
Mastectomy	11122 (46.6)	11142 (46.7)	
*AJCC_Stage*			
I	10718 (44.9)	10698 (44.8)	0.123
II	8622 (36.1)	8817 (36.9)	
III	3631 (15.2)	3503 (14.7)	
IV	891 (3.7)	844 (3.5)	
*AJCC_T*			
1	12259 (51.4)	12249 (51.3)	<0.001
2	8214 (34.4)	8066 (33.8)	
3	2568 (10.8)	3048 (12.8)	
4	821 (3.4)	499 (2.1)	
*AJCC_N*			
0	15296 (64.1)	15377 (64.4)	0.133
1	5995 (25.1)	5946 (24.9)	
2	1457 (6.1)	1360 (5.7)	
3	1114 (4.7)	1179 (4.9)	
*AJCC_M*			
0	22968 (96.3)	23015 (96.5)	0.261
1	894 (3.7)	847 (3.5)	
*Subtype*			
HR−/HER2−	342 (1.4)	343 (1.4)	0.977
HR−/HER2+	108 (0.5)	109 (0.5)	
HR+/HER2−	22343 (93.6)	22361 (93.7)	
HR+/HER2+	1069 (4.5)	1049 (4.4)	
*ER_Status*			
Negative	480 (2.0)	488 (2.0)	0.82
Positive	23382 (98.0)	23374 (98.0)	
*PR_Status*			
Negative	3983 (16.7)	3972 (16.6)	0.902
Positive	19879 (83.3)	19890 (83.4)	
*HER2_Status*			
Negative	22685 (95.1)	22704 (95.1)	0.702
Positive	1177 (4.9)	1158 (4.9)	
*Radiation*			
Yes	11859 (49.7)	11734 (49.2)	0.256
No	12003 (50.3)	12128 (50.8)	
*Chemotherapy*			
Yes	16517 (69.2)	16558 (69.4)	0.691
No	7345 (30.8)	7304 (30.6)	

ILC: Invasive lobular carcinoma; IDC: Invasive ductal carcinoma.

## Results

### Patient characteristics between ILC and IDC

Patient characteristics differed between ILC and IDC patients. Using the SEER tumor registry database, we identified 879,718 patients diagnosed with either ILC or IDC. By applying specific inclusion and exclusion criteria, we narrowed our sample to 224,054 patients. These patients were then divided into two groups: 23,862 (10.7%) in the ILC group and 200,192 (89.3%) in the IDC group.

The clinical characteristics of the ILC and IDC groups are summarized in Table 1. The ILC patients, in comparison to IDC patients, were found to be older, have more advanced tumor stage, larger tumor size, higher incidence of axillary lymph node metastasis, greater positivity of ER and PR receptors, lower incidence of HER2 positivity, and were less likely to receive chemotherapy (*P* < 0.001 for all variables). When comparing the surgical procedures, it was found that ILC cases had a higher percentage of mastectomy in comparison to IDC cases (48.0% compared to 36.7%, respectively). Additionally, the ILC group had a higher rate of receiving radiation therapy and a lower rate of receiving chemotherapy (51.5% compared to 47.8% and 31.4% compared to 57.6%, respectively). This difference was statistically significant (*P* < 0.001).

### Survival outcomes between ILC and IDC groups

**Figure 1.**
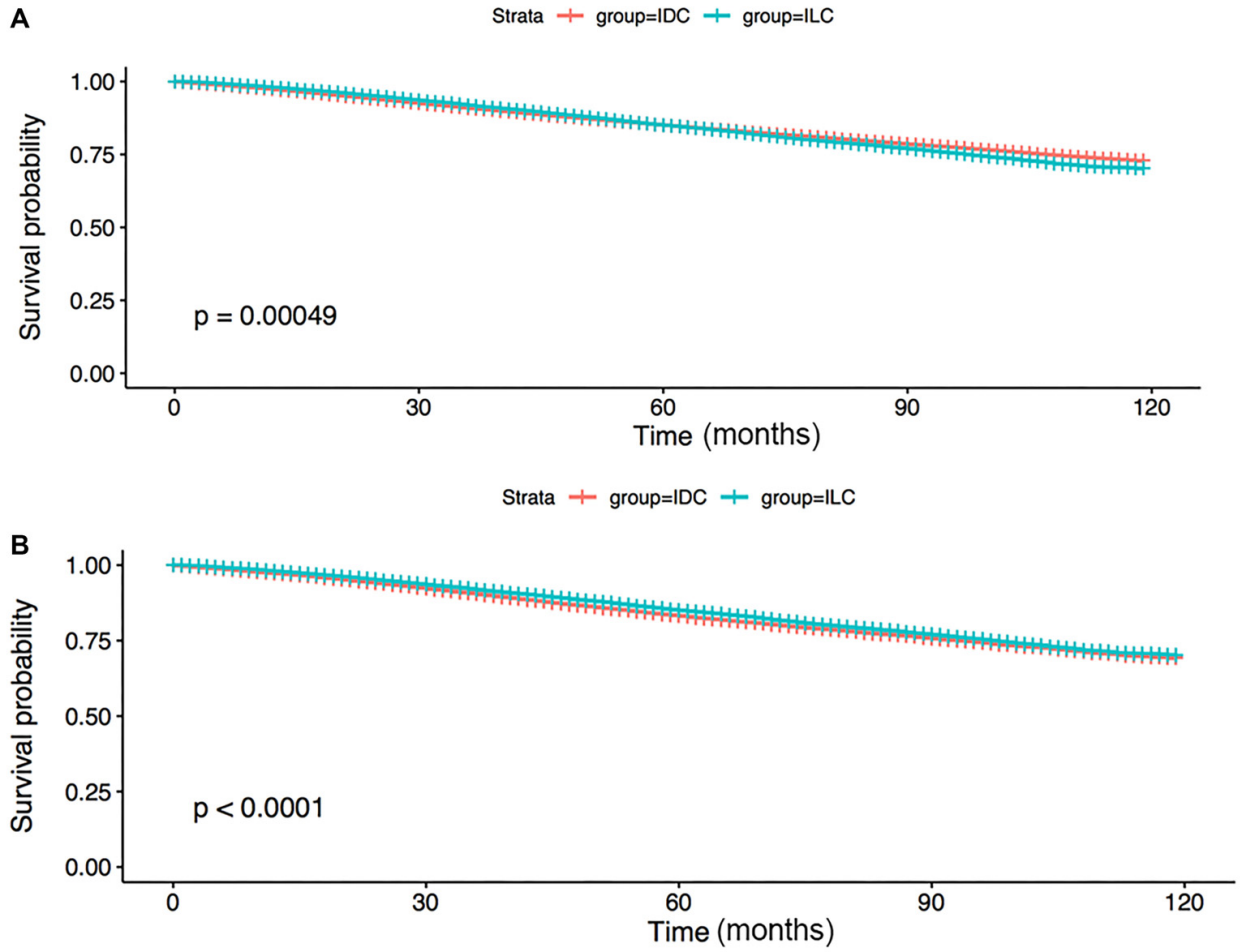
(A) OS curves of all unmatched IDC and ILC patients; (B) OS curves of all matched IDC and ILC patients. OS: Overall survival; ILC: Invasive lobular carcinoma; IDC: Invasive ductal carcinoma.

Given the significant inter-group disparities in clinical traits, the PSM technique was adopted based on race, age, laterality, primary site, surgery, TNM phase, subtype, radiation, chemotherapy, as well as statuses of HER2, ER, and PR, for lowering the intergroup disparities in survival outcomes. We matched every ILC patient to one IDC patient. According to Table 2, the two groups were constituted by patients in a 1:1 ratio with resembling baseline clinicopathological traits for subsequent analysis. Figure 1A displayed the OS in patients with ILC compared to those with IDC in the unmatched population. The prognosis for ILC seemed to be better than that for IDC in the first five years after diagnosis. However, after 5–10 years, ILC patients showed a worse prognosis. In contrast, after matching with the PSM technique, the ILC group demonstrated significantly better OS (*P* < 0.001) compared to the IDC group (Figure 1B). Interestingly, the survival curves appeared to merge after a long-term follow-up.

### Independent prognostic factors in ILC

**Figure 2.**
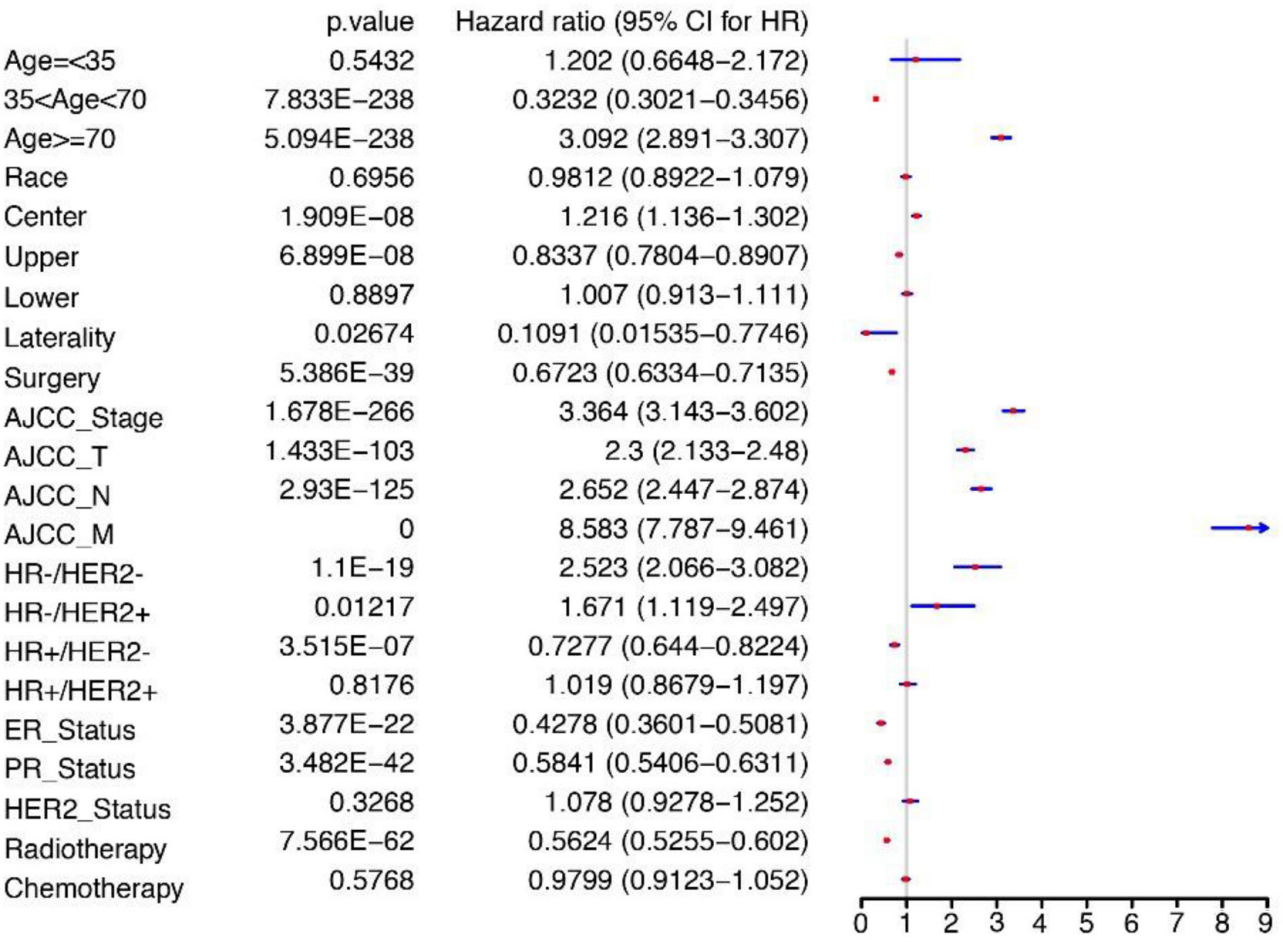
**Univariate Cox analysis for OS of ILC patients.** OS: Overall survival; ILC: Invasive lobular carcinoma.

**Figure 3.**
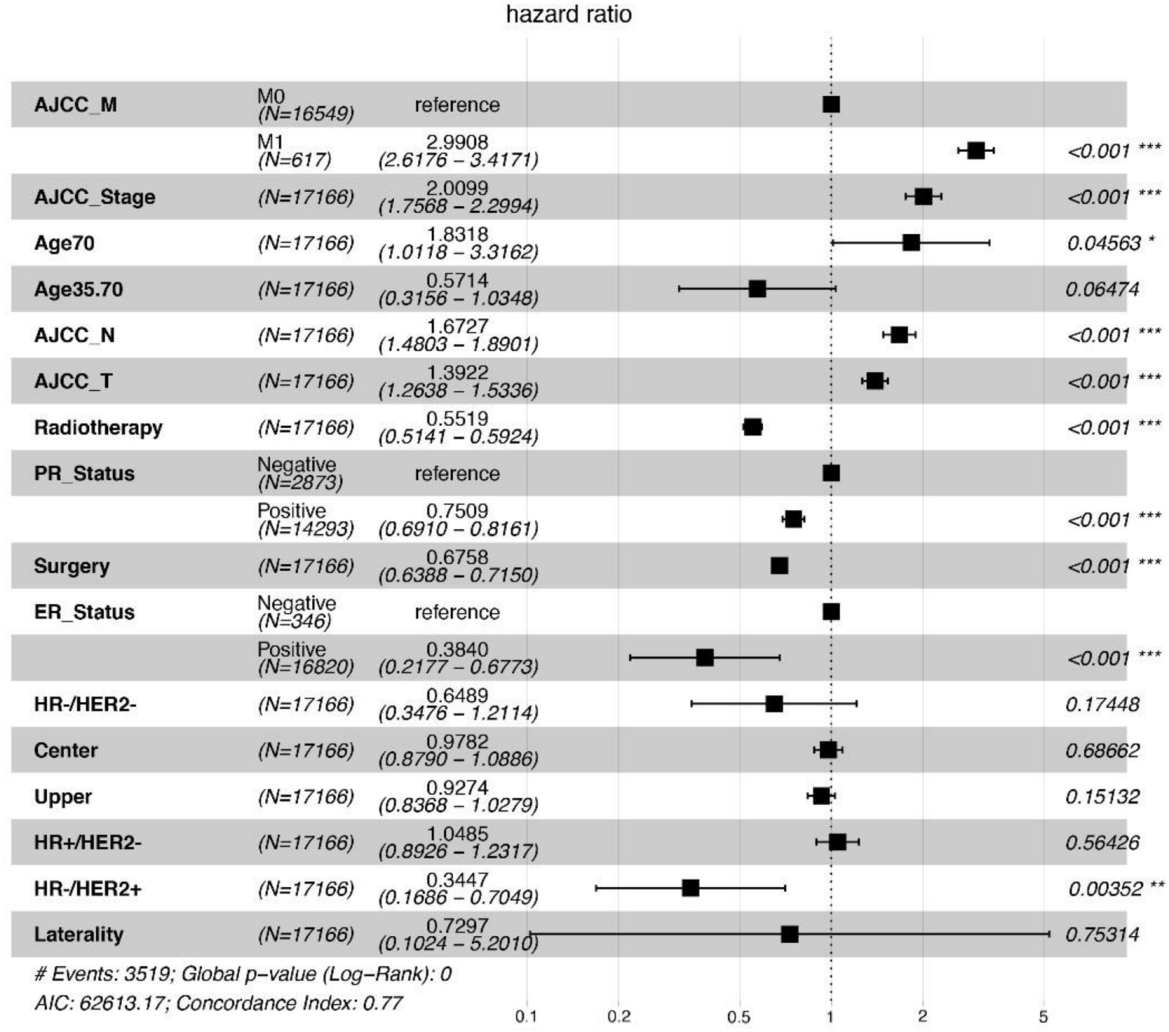
**Forest plot of OS for ILC patients.** OS: Overall survival; ILC: Invasive lobular carcinoma.

Based on the univariate Cox regression analysis for OS in the training cohort, several factors were identified as significant prognostic indicators, including age, primary site, laterality, surgery, the extent of T, N, and M, TNM stages, ER, PR, breast subtype, and radiotherapy. These variables were deemed statistically significant with *P* values less than 0.05 and demonstrated reasonable HR values (Figure 2). To further investigate the relationship between tumor characteristics and survival outcomes, a multivariate Cox regression model was utilized. The above indicators were then subjected to multivariate analysis, revealing T, N and M stages, TNM stage, age > 70, radiotherapy, surgery, PR, ER, and HR−/HER2+ as independent predictors of OS for the ILC group (Figure 3).

### Prognostic nomogram for survival

**Figure 4.**
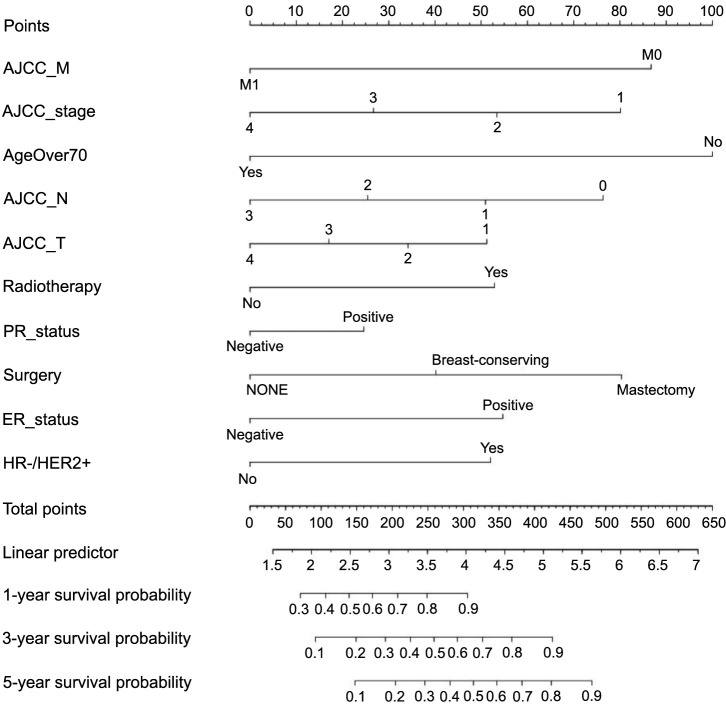
**Nomogram to predict 1-, 3-, and 5-year OS of ILC patients**. Notes: Vertical line between each variable and points scale can be drawn to acquire points of each variable. Predicted survival rate was calculated according to the total points by drawing a vertical line from Total Points scale to OS scale. OS: Overall survival; ILC: Invasive lobular carcinoma.

Using the multivariate Cox results obtained from our training cohort, we created predictive nomograms for 1-, 3-, and 5-year OS. These nomograms included all independent prognostic indicators (Figure 4). Our model showed that advanced age had the greatest impact on prognosis, followed by the presence of metastases and whether surgery was performed. Other factors, such as stage, T and N stages, PR and ER status, radiotherapy, and HER-2 status, had a moderate effect on OS. Given the correspondence of every parameter in the nomogram to a score by the multivariate Cox regression-derived weight, our formulated nomogram was interpretable. An overall risk score was yielded for every patient by summing up the entire parameter scores, thereby enabling OS inference. The particular procedure of nomogram interpretation has been described before [18].

**Figure 5.**
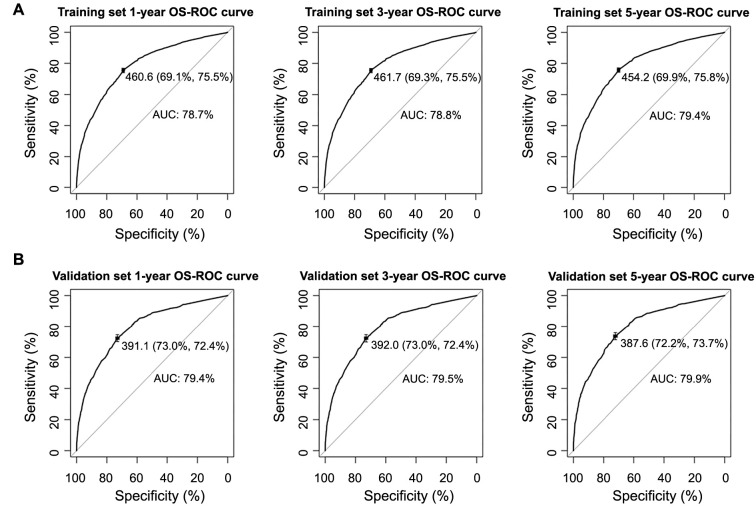
**The ROC curve and AUC.** (A) Predicting 1-, 3-, and 5-year OS in the training cohort; (B) Predicting 1-, 3-, and 5-year OS in the validation cohort. ROC: Receiver operating characteristics; AUC: Area under the ROC curve; OS: Overall survival.

When computing nomograms, the scores for different risk predictors, such as being 45 years old (100), having HR−/HER2+ breast cancer (50), being T2N1M0 (170), undergoing breast-conserving surgery (40), and receiving radiotherapy (50), would be taken into account for a 45-year-old woman with HR+/HER2+ breast cancer who has had breast-conserving surgery and radiotherapy. This would yield an overall score of 410. Our model predicts an 85% probability of the woman surviving for three years and a 75% probability at five years.

### Performance and validation of the nomogram

**Figure 6.**
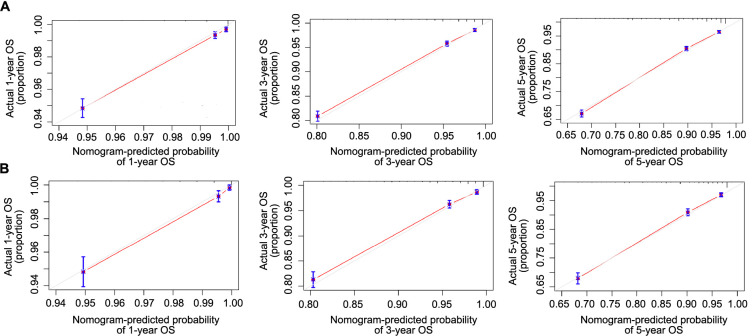
**The calibration curves to predict 1-, 3-, and 5-year OS in the training set (A) and the internal validation set (B).** OS: Overall survival.

**Figure 7.**
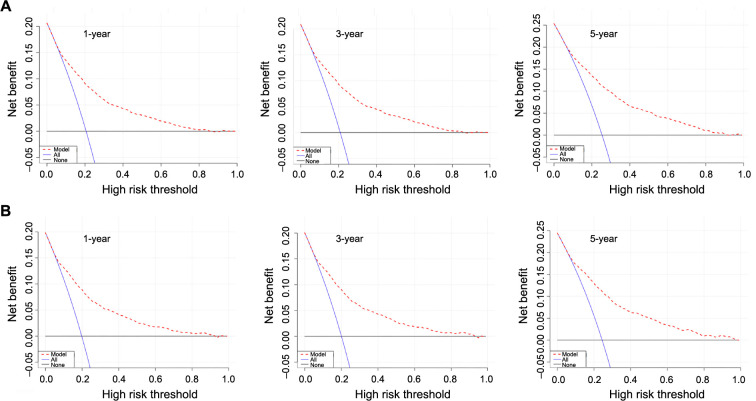
**DCA curves of the nomogram for predicting 1-, 3- and 5-year OS in the training set (A) and the internal validation set (B)**. DCA: Decision curve analysis; OS: Overall survival.

The calibration curves of the nomogram showed strong consistency in predicting OS for both the training set (Figure 5A) and the internal validation set (Figure 5B). The nomogram achieved a C-index of 0.776 for predicting OS in the training cohort. Additionally, the area under the ROC curve (AUC) at one year was 0.787, at three years was 0.788, and at five years was 0.794. In the validation cohort, the predicted OS C-index was 0.785. The AUC values at one year, three years, and five years were 0.794, 0.795, and 0.799, respectively. The calibration curves (Figure 6) demonstrated that the data points were closely aligned with the 45∘ diagonal line, indicating highly accurate predictive capabilities of the nomogram. To compare the clinical usefulness of the nomogram with the traditional AJCC staging system, DCA was conducted. The DCA curves (Figure 7) revealed that the nomogram had superior predictive abilities for 1-, 3-, and 5-year OS, potentially resulting in greater clinical benefits.

## Discussion

Firstly, we obtained data on 200,192 patients diagnosed with IDC and 23,862 patients diagnosed with ILC from the SEER database. We observed that individuals with ILC were typically diagnosed at a later age, had larger tumor sizes, and a higher expression of ER/PR, and were less likely to undergo radiation therapy or chemotherapy. During the initial five years post-diagnosis, the prognosis for patients with ILC was more favorable compared to those with IDC. However, from 5 to 10 years, ILC patients exhibited poorer prognoses. These findings align with previous research [19].

Secondly, to ensure that differences in survival outcomes are not influenced by variations in baseline clinical characteristics, we employed the PSM method to perform a 1:1 case-control analysis comparing ILC and IDC patients. The resulting matched data showed that patients with ILC had a more favorable prognosis than those with IDC. The existing literature on the prognoses of ILC vs IDC presents conflicting views. Some studies suggest that ILC has a better prognosis [20], whereas others reported similar prognoses [10, 21–23]. Other studies demonstrated that the prognosis for ILC is worse than IDC [8, 24–26]. This discrepancy in the literature may arise from the fact that ILC represents a diverse group of tumors with outcomes closely related to the specific histological variant [27], consequently, aggregating all ILC cases together leads to varying results depending on the prevalence of each variant.

Thirdly, the findings from the current study indicate that age is a significant independent factor in predicting OS. Consistent with previous research, older patients have a higher risk of poor outcomes [21]. One possible explanation for this is that older individuals are more susceptible to multiple health conditions, which increases their risk. This suggests that providing treatment solely for ILC may not be sufficient for older patients, and their co-existing conditions should also be addressed. In addition, the results confirm that tumor stage (T, N, and M) is an important prognostic factor for breast cancer patients [28]. Furthermore, the study found that HR and HER2 status were independent predictors of OS. Typically, classic ILC presents with a luminal A molecular subtype, with a higher proportion of cases showing strong expression of ER and PR compared to IDC [7, 29]. ILC also commonly lacks the expression of HER2 [29]. A recent study was conducted on Mexican breast cancer patients comparing the DFS and OS rates between ILC and IDC. The study revealed that the OS rates for both triple-negative ILC and HER2-positive ILC were significantly worse compared to IDC [26, 30]. The study also compared HER2-positive ILC and HER2-positive IDC patients, providing further evidence that HER2-positive ILC exhibits distinct clinical and biological characteristics relative to HER2-positive IDC [31]. Specifically, HER2-positive ILCs were more likely to be multicentric or multifocal, had a lower histological grade and proliferative index, and showed a higher frequency of nodal metastases [31]. Despite these differences, both HER2-positive ILC and IDC patients appeared to benefit similarly from adjuvant treatment with trastuzumab, resulting in similar recurrence rates. This suggests that HER2-positive ILC patients do derive benefits from anti-HER2 therapy [32, 33].

Fourthly, this study found that surgery and radiotherapy were effective in reducing the risk of death, which aligns with previous findings [5, 34, 35]. Similar benefits have been observed in smaller studies conducted at single institutions and in larger population-based analyses, indicating a reduction in local regional recurrence and improved survival rates [35]. However, the incidence of death was not affected by receiving chemotherapy. It is known that ILC generally shows a poorer response to adjuvant chemotherapy compared to IDC [36, 37]. This may reflect the fact that around 90% of ILCs are Luminal A tumors, exhibiting low histologic grades and low mitotic indices, thus limiting their responses to chemotherapy, while the high mastectomy rate can be attributed to relatively larger tumor size [38].

Finally, our nomogram encompassed a comprehensive range of clinical risk factors that can easily be obtained from historical records. These factors include age, race, laterality, primary site, surgery, TNM phase, subtype, radiation, chemotherapy, as well as statuses of HER2, ER, and PR. As indicated by the preferable fitting of calibration graphs and comparatively high C-indices, our nomogram performs strongly. Furthermore, this nomogram is user-friendly, since a point score is assigned to every trait at its top, and the overall score can be derived through a simple summation of the entire individual item scores. A vertical line plotted from the overall score at the nomogram bottom intersects with three lines, indicating the cumulative incidence risks of death at one, three, and five years for the patients.

Our research has a few shortcomings. At first, it was impossible to differentiate “pure” ILC from “hybrid” ILC across various geographic locations in the SEER database. Prognoses vary by the histological subclass of ILC, with pleomorphic ILC exhibiting more aggressive clinical traits and an inferior outcome compared to common ILC [39]. This study had inherent selection biases and data deficiencies due to its retrospective cohort design based on the SEER registries. Second, this study only had internal validation and lacked external validation. While we are currently collecting follow-up data from our hospital’s breast lobular carcinoma patients for external validation, the current number of patients is inadequate.

## Conclusion

 We will continue to collect and analyze the follow-up data of ILC patients. There is a scarcity of extensive and up-to-date data sets allowing for a thorough contrast of clinicopathologic traits between ILC and IDC. Therefore, our research utilized the SEER database to conduct a comprehensive analysis of ILC patients and develop a nomogram to predict the 1-, 3-, and 5-year OS rates. This model can be beneficial to clinicians in evaluating the prognosis of ILC and assisting in decision making and patient counseling.
